# Associations Between Hand Hygiene Education and Self-Reported Hand-Washing Behaviors Among Korean Adults During MERS-CoV Outbreak

**DOI:** 10.1177/1090198118783829

**Published:** 2018-07-16

**Authors:** Jieun Yang, Eun-Cheol Park, Sang Ah Lee, Sang Gyu Lee

**Affiliations:** 1 Yonsei University, Seoul, Republic of Korea

**Keywords:** hand-washing education, hand-washing promotion, MERS outbreak, self-reported hand washing with sanitizer, self-reported hand washing with soap, self-reported hand-washing frequency

## Abstract

*Background.* Hand washing is an effective way to prevent transmission of infectious diseases. Education and promotional materials about hand washing may change individuals’ awareness toward hand washing. Infectious disease outbreak may also affect individuals’ awareness. *Aims.* Our study aimed to examine associations between hand-washing education and self-reported hand-washing behaviors among Korean adults during the year of the Middle East respiratory syndrome (MERS) outbreak. *Methods.* Data from the 2015 Community Health Survey were used for this study. The total study population comprised 222,599 individuals who were older than 20 years of age. A multiple linear regression model was used to investigate associations between hand hygiene education and self-reported hand-washing behaviors. Subgroup analyses stratified by age, sex, income, and MERS outbreak regions were also performed. *Results.* Individuals who received hand-washing education or saw promotional materials related to hand washing had significantly higher scores for self-reported use of soap or sanitizer (β = 0.177, *P* < .0001) and self-reported frequency of hand washing (β = 0.481, *P* < .0001) than those who did not have such experiences. The effect of hand-washing education on self-reported behavior change was greater among older adults, women, and lower income earners. The effect of hand hygiene education on self-reported use of soap or sanitizer was similar regardless of whether the participants lived in MERS regions. *Conclusion.* Our findings emphasize the importance of education or promotions encouraging hand washing, especially for older adults, women, and lower income earners. In addition, MERS outbreak itself affected individuals’ awareness of hand-washing behaviors. Well-organized campaigns that consider these factors are needed to prevent infectious diseases.

Hand washing has long been considered an effective way to prevent transmission of infectious diseases, such as respiratory and gastrointestinal infections, trachoma, and worm infections (Biran et al., 2014). Moreover, hand washing with soap or hand sanitizer reduces infection rates compared with hand washing with only water (Burton et al., 2011; Fendler et al., 2002; Luby et al., 2005; Paulson, Fendler, Dolan, & Williams, 1999; Sandora et al., 2005; Zaragoza, Sallés, Gomez, Bayas, & Trilla, 1999). Previous studies in other countries found that hand hygiene education effectively increased health care workers’ hand-washing compliance (Mathai et al., 2010; Rosenthal, McCormick, Guzman, Villamayor, & Orellano, 2003). A hand-washing promotion or campaign was also effective in increasing individuals’ awareness and performance of hand hygiene practices (Randle, Clarke, & Storr, 2006).

In May 2015, a severe outbreak of Middle East respiratory syndrome (MERS) occurred in several regions of South Korea (hereafter, Korea). During the outbreak, 186 confirmed cases were reported across the country (Korean Centers for Disease Control and Prevention [KCDC], 2017b). MERS is an acute respiratory disease that is caused by a newly identified strain of coronavirus, Middle East respiratory syndrome coronavirus (World Health Organization, 2017). At this point, there is no effective vaccine against this worldwide disease, whose fatality rate is approximately 35% (World Health Organization, 2017). Proper hand washing is considered the first and most crucial means of preventing MERS infection (KCDC, 2017b). Therefore, the MERS outbreak may have raised Koreans’ awareness of the importance of hand hygiene and changed hand-washing behavior.

Previous studies in Korea have focused on hand hygiene performance rates among health care workers (Oh, 2015), elementary school to high school students (Yang et al., 2014), and adults (Y.-H. Lee et al., 2016). Other studies have investigated the relationship between hand hygiene education and the behaviors of targeted individuals, including nurses (Kim & Choi, 2002), undergraduate students (Choi, Jang, & Choi, 2014), and adolescents (Min & Chang, 2014). Additionally, one study focused on the National Handwashing Survey administered to Korean adults from 2006 to 2014 (M.-S. Lee, Hong, & Kim, 2015); however, this study was conducted before the 2015 MERS outbreak in Korea.

Therefore, the present study aimed to examine associations of hand-washing education with self-reported hand-washing frequency and self-reported use of soap or hand sanitizer among Korean adults during the year of the MERS outbreak in Korea using a nationally representative survey conducted 3 months after the first confirmed case occurred. We also performed subgroup analyses to evaluate associations of hand hygiene education or promotion with self-reported hand hygiene frequency and self-reported use of soap or hand sanitizer according to age group, sex, income, and MERS outbreak region.

## Method

### Study Population

This study analyzed data from the Community Health Survey (CHS) conducted by the KCDC. The CHS has been conducted annually since 2008 among individuals aged 19 years and older across the country. We used data from the 2015 CHS. The study’s population was limited to adults ≥20 years old, and we excluded individuals who were younger than 20 years old (*n* = 2,616) and individuals with missing data (*n* = 3,343). Therefore, the present study evaluated data from 222,599 individuals. The CHS received ethical approval from the institutional review board of the KCDC (IRB No. 2014-08EXP-09-4C-A), and written consent was obtained from all survey participants.

### Variables

The main variable of interest was hand-washing education and promotion, which was assessed using self-reported data based on responses to the CHS question, “Did you receive any education on correct hand washing or see promotional materials related to hand washing during the past year?” Possible responses were “yes” and “no.”

The dependent variables were self-reported hand-washing method and frequency. These were evaluated using self-reported data based on responses to the CHS questionnaire. To evaluate hand-washing methods, we used the question, “How often do you usually wash your hands with soap or hand sanitizer?” The possible responses were “always,” “often,” “sometimes,” “rarely,” and “never.” Hand-washing method was scored on a 5-point scale from 1 (*never*) to 5 (*always*). To evaluate hand-washing frequency, we used three questions: “How often did you wash your hands before having a meal during the past week?” “How often did you wash your hands after using the toilet during the past week?” “How often did you wash your hands after returning from outside during the past week?” Each question had four possible responses: “always,” “often,” “sometimes,” and “rarely.” Hand-washing frequency in each situation was scored on a 4-point scale from 1 (*rarely*) to 4 (*always*). The total score for hand-washing frequency was the sum of the three questions; thus, total scores ranged from 3 to 12.

The analyses were adjusted for participants’ demographic, socioeconomic, and health-related characteristics. The demographic characteristics considered were age group (20-29, 30-39, 40-49, 50-59, 60-69, 70-79, or ≥80 years), sex (male or female), and marital status (married and cohabitating, married and non-cohabitating, or single). The socioeconomic characteristics were income level (low, lower middle, upper middle, or high), education (elementary school and less, middle school, high school, or college and higher), and occupation (white collar [managerial, professional, or clerical], pink collar [sales and services], blue collar [manual labor], or nonworking). The health-related factor was perceived health status (good, average, or poor). We also adjusted for residence in a MERS outbreak region in Korea (yes or no), as MERS occurred in 43 of the 254 regions where the 2015 CHS was conducted (”List of hospitals,” 2017). All covariates were treated as categorical variables.

### Statistical Analysis

The general characteristics of the study participants were examined using *t* tests and analysis of variance. A multiple linear regression model was used to investigate associations of hand hygiene education with self-reported hand-washing behaviors, including both methods and frequency, among Korean adults after adjusting for demographic, socioeconomic, and health-related characteristics. Subgroup analyses were also performed, with participants stratified by age, sex, income, and MERS outbreak regions. Differences were considered statistically significant at *p* values <.05. Effect sizes were calculated using partial eta squared, which refers to the proportion of variance explained. All statistical analyses were performed using SAS software (version 9.4, SAS Institute, Cary, NC).

## Results

Table 1 shows the general characteristics of the study population. Among the 222,599 participants, 169,662 (76.2%) indicated that they had received education on correct hand washing or had seen hand-washing–related promotional materials within the past year, whereas 52,937 participants (23.8%) reported no exposure to hand-washing education or promotional materials on hand washing. The means and standard deviations (*SD*s) for hand washing with soap or hand sanitizer were 4.23 ± 0.90 among those who had and 3.87 ± 1.04 among those who had not experienced hand-washing education, respectively, and the means and *SD*s for hand-washing frequency were 10.36 ± 1.81 and 9.54 ± 2.14, respectively.

**Table 1.** General Characteristics of Study Population.

Variables	Total (*N* = 222,599)		Self-reported hand hygiene behavior							
			Using soap or hand sanitizer				Hand-washing frequency			
	*N*	%	*M*	*SD*	*P* value	ES^a^	*M*	*SD*	*P* value	ES^a^
Hand hygiene education or promotion					<.0001	0.006			<.0001	0.012
Yes	169,662	76.2	4.23	0.90			10.36	1.81		
No	52,937	23.8	3.87	1.04			9.54	2.14		
Age (years)					<.0001	0.007			<.0001	0.007
20-29	21,796	9.8	4.40	0.80			10.32	1.77		
30-39	32,077	14.4	4.47	0.76			10.58	1.68		
40-49	41,305	18.6	4.30	0.86			10.37	1.81		
50-59	44,725	20.1	4.15	0.93			10.25	1.86		
60-69	37,800	17.0	4.04	0.99			10.15	1.93		
70-79	32,673	14.7	3.80	1.03			9.71	2.10		
≥80	12,223	5.5	3.55	1.05			9.07	2.30		
Sex					<.0001	0.003			<.0001	0.048
Male	100,136	45.0	4.12	0.97			9.73	2.08		
Female	122,463	55.0	4.17	0.94			10.52	1.72		
Marital status					<.0001	0.000			<.0001	0.001
Married, cohabiting	153,763	69.1	4.17	0.94			10.24	1.90		
Married, non-cohabiting	37,656	16.9	3.87	1.02			9.85	2.05		
Single	31,180	14.0	4.35	0.84			10.18	1.87		
Income					<.0001	0.001			<.0001	0.000
Low	48,530	21.8	3.80	1.05			9.68	2.13		
Lower-middle	79,320	35.6	4.15	0.94			10.13	1.93		
Upper-middle	60,095	27.0	4.30	0.86			10.38	1.79		
High	34,654	15.6	4.37	0.83			10.55	1.72		
Education					<.0001	0.010			<.0001	0.009
Elementary school or less	59,490	26.7	3.75	1.03			9.65	2.11		
Middle school	24,591	11.1	4.05	0.97			9.99	1.99		
High school	72,554	32.6	4.26	0.89			10.23	1.87		
College or over	65,964	29.6	4.42	0.80			10.62	1.66		
Occupation					<.0001	0.001			<.0001	0.002
White collar	43,313	19.5	4.42	0.79			10.62	1.63		
Pink collar	29,159	13.1	4.28	0.87			10.48	1.72		
Blue collar	70,084	31.5	4.01	0.98			9.82	2.00		
Nonworking	80,043	36.0	4.07	0.99			10.11	2.02		
Perceived health status					<.0001	0.002			<.0001	0.006
Good	81,931	36.8	4.28	0.88			10.41	1.79		
Average	92,838	41.7	4.18	0.93			10.22	1.87		
Poor	47,830	21.5	3.84	1.04			9.65	2.16		
MERS outbreak region					<.0001	0.001			<.0001	0.001
Yes	37,886	17.0	4.29	0.88			10.42	1.79		
No	184,713	83.0	4.12	0.96			10.11	1.95		
Total	222,599	100.0	4.15	0.95			10.17	1.93		

*Note.* ES = effect size; MERS = Middle East respiratory syndrome.

a Effect sizes were computed using partial eta-squared.

Table 2 shows the factors associated with self-reported hand-washing methods and frequency. The results demonstrate that exposure to hand-washing education or promotional materials was significantly associated with self-reported hand-washing behaviors. Individuals who had received education on correct hand washing or seen promotional materials related to hand washing within the previous year had significantly higher scores for both self-reported hand-washing methods (β = 0.177, *P* < .0001) and self-reported hand-washing frequency (β = 0.481, *P* < .0001) than those who had not had such educational opportunities. Men exhibited significantly lower scores for both self-reported hand-washing methods (β = −0.113, *P* < .0001) and self-reported hand-washing frequency (β = −0.900, *P* < .0001) than women. With the highest income level set as the reference group, scores for self-reported hand-washing methods (β = −0.030, *P* < .0001; β = −0.051, *P* < .0001; β = −0.131, *P* < .0001) and self-reported frequency (β = −0.063, *P* < .0001; β = −0.083, *P* < .0001; β = −0.122, *P* < .0001) were progressively lower in the following order: upper middle > lower middle > low income, respectively. Individuals who lived in MERS outbreak regions showed significantly higher scores for both self-reported hand-washing methods (β = 0.081, *P* < .0001) and self-reported hand-washing frequency (β = 0.154, *P* < .0001) than did those who lived elsewhere.

**Table 2.** Factors Associated With Self-Reported Hand Hygiene Behavior (Methods, Frequency).

Variables	Self-reported hand hygiene behavior					
	Using soap or hand sanitizer			Hand-washing frequency		
	β	*SE*	*P* value	β	*SE*	*P* value
Hand hygiene education or promotion						
Yes	0.177	0.005	<.0001	0.481	0.009	<.0001
No	Ref.			Ref.		
Age (years)						
20-29	0.422	0.014	<.0001	0.472	0.028	<.0001
30-39	0.442	0.012	<.0001	0.560	0.024	<.0001
40-49	0.307	0.012	<.0001	0.435	0.023	<.0001
50-59	0.269	0.011	<.0001	0.548	0.022	<.0001
60-69	0.302	0.010	<.0001	0.743	0.020	<.0001
70-79	0.197	0.010	<.0001	0.543	0.020	<.0001
≥80	Ref.			Ref.		
Sex						
Male	−0.113	0.004	<.0001	−0.900	0.009	<.0001
Female	Ref.			Ref.		
Marital status						
Married, cohabiting	0.055	0.008	<.0001	0.221	0.016	<.0001
Married, non-cohabiting	0.033	0.010	0.0005	0.106	0.019	<.0001
Single	Ref.			Ref.		
Income						
Low	−0.131	0.008	<.0001	−0.122	0.016	<.0001
Lower-middle	−0.051	0.006	<.0001	−0.083	0.012	<.0001
Upper-middle	−0.030	0.006	<.0001	−0.063	0.012	<.0001
High	Ref.			Ref.		
Education						
Elementary school or less	−0.358	0.008	<.0001	−0.718	0.016	<.0001
Middle school	−0.182	0.008	<.0001	−0.498	0.016	<.0001
High school	−0.068	0.005	<.0001	−0.280	0.011	<.0001
College or over	Ref.			Ref.		
Occupation						
White collar	0.017	0.006	0.0095	0.143	0.013	<.0001
Pink collar	0.001	0.007	0.9334	0.099	0.013	<.0001
Blue collar	−0.071	0.005	<.0001	−0.098	0.010	<.0001
Nonworking	Ref.			Ref.		
Perceived health status						
Good	0.116	0.006	<.0001	0.423	0.012	<.0001
Average	0.087	0.006	<.0001	0.253	0.011	<.0001
Poor	Ref.			Ref.		
MERS outbreak region						
Yes	0.081	0.005	<.0001	0.154	0.010	<.0001
No	Ref.			Ref.		

*Note. SE* = standard error; MERS = Middle East respiratory syndrome. Multiple linear regressions were performed.

Table 3 shows the results of subgroup analyses stratified by age group, sex, income, and MERS outbreak region. The subgroup analyses showed significant differences in each group, although modifying effects of sex on self-reported use of soap or sanitizer and MERS outbreak region on both dependent variables were not significant in the tests for interaction. Participants in the 30 to 39, 40 to 49, 50 to 59, 60 to 69, 70 to 79, and ≥80 years age groups who had received education on correct hand washing or had seen related promotional materials exhibited higher scores with age for both self-reported hand-washing methods (β = 0.090, *P* < .0001; β = 0.119, *P* < .0001; β = 0.161, *P* < .0001; β = 0.198, *P* < .0001; β = 0.239, *P* < .0001; and β = 0.319, *P* < .0001, respectively) and self-reported hand-washing frequency (β = 0.267, *P* < .0001; β = 0.387, *P* < .0001; β = 0.439, *P* < .0001; β = 0.471, *P* < .0001; β = 0.603, *P* < .0001; and β = 0.830, *P* < .0001, respectively). Women who received education on correct hand washing or saw promotional materials related to hand washing showed higher scores in self-reported hand-washing methods (β = 0.211, *P* < .0001) than did men. Women who received education on hand washing or saw promotional materials showed a trend toward a greater magnitude of self-reported hand-washing frequency scores than men. As income level decreased from high to upper middle, lower middle, and low, individuals who reported exposure to hand-washing education or promotional materials exhibited progressively higher scores in both self-reported hand-washing methods (β = 0.111, *P* < .0001; β = 0.131, *P* < .0001; β = 0.169, *P* < .0001; β = 0.247, *P* < .0001, respectively) and self-reported hand-washing frequency (β = 0.373, *P* < .0001; β = 0.397, *P* < .0001; β = 0.480, *P* < .0001; β = 0.586, *P* < .0001, respectively). Individuals who lived in MERS outbreak regions and who had exposure to hand-washing education or promotional materials showed a trend toward a slightly greater magnitude of self-reported hand-washing frequency scores than individuals who did not live in those regions (β = 0.528, *P* < .0001; β = 0.472, *P* < .0001, respectively).

**Table 3.** Subgroup Analysis of the Association Between Hand Hygiene Education and Self-Reported Hand Hygiene Behavior (Methods, Frequency).

Variables	Self-reported using soap or hand sanitizer				Self-reported hand-washing frequency			
	Education				Education			
	Yes			No	Yes			No
	β	*SE*	*P* value	β	β	*SE*	*P* value	β
Age (years)								
20-29	0.078	0.014	<.0001	Ref.	0.339	0.030	<.0001	Ref.
30-39	0.090	0.012	<.0001	Ref.	0.267	0.025	<.0001	Ref.
40-49	0.119	0.012	<.0001	Ref.	0.387	0.023	<.0001	Ref.
50-59	0.161	0.011	<.0001	Ref.	0.439	0.021	<.0001	Ref.
60-69	0.198	0.012	<.0001	Ref.	0.471	0.022	<.0001	Ref.
70-79	0.239	0.012	<.0001	Ref.	0.603	0.023	<.0001	Ref.
≥80	0.319	0.019	<.0001	Ref.	0.830	0.040	<.0001	Ref.
Sex								
Male	0.144	0.007	<.0001	Ref.	0.447	0.015	<.0001	Ref.
Female	0.211	0.006	<.0001	Ref.	0.511	0.012	<.0001	Ref.
Income								
Low	0.247	0.010	<.0001	Ref.	0.586	0.019	<.0001	Ref.
Lower-middle	0.169	0.008	<.0001	Ref.	0.480	0.016	<.0001	Ref.
Upper-middle	0.131	0.009	<.0001	Ref.	0.397	0.019	<.0001	Ref.
High	0.111	0.012	<.0001	Ref.	0.373	0.025	<.0001	Ref.
MERS outbreak region								
Yes	0.174	0.011	<.0001	Ref.	0.528	0.022	<.0001	Ref.
No	0.177	0.005	<.0001	Ref.	0.472	0.010	<.0001	Ref.

*Note. SE* = standard error; MERS = Middle East respiratory syndrome.

## Discussion

The results of the present study revealed that self-reported behaviors related to hand-washing methods and frequency were significantly associated with exposure to hand-washing education and promotion. Individuals who had received education on correct hand washing or saw related promotional materials within the previous year had higher scores in both self-reported hand-washing methods and self-reported hand-washing frequency than did those who did not have such experiences. Men, single people, individuals with lower incomes, and individuals who had less education had significantly lower scores for both self-reported hand-washing methods and self-reported hand-washing frequency than did their counterparts. The associations reported here are similar to those in previous studies on hand-washing behaviors in several countries, including associations with hand hygiene education (Pittet et al., 2000), sex (Van de Mortel, Bourke, McLoughlin, Nonu, & Reis, 2001), income, and educational level (Y.-H. Lee et al., 2016). Interestingly, in our study, individuals who lived in MERS outbreak regions showed significantly higher scores for both self-reported hand-washing methods and self-reported hand-washing frequency than did those who lived elsewhere. In Korea, the first confirmed MERS patient occurred on May 20, 2015, and the number of confirmed patients rapidly increased in June 2015. As of July 4, 2015, no more confirmed cases were reported (Ministry of Health and Welfare, 2016). Meanwhile, the 2015 CHS that we used in this study was performed from August 31, 2015, to November 8, 2015 (KCDC, 2017a). Since the survey was conducted only a few months after the severe outbreak, the responses to the survey questions were probably affected by individuals’ motivation to prevent the disease. A major outbreak of severe acute respiratory syndrome (SARS), which is mainly transmitted by respiratory droplets, occurred in 2002 in Hong Kong. The SARS fatality rate was high, and it served to remind individuals living in Hong Kong of the importance of hand washing; indeed, their self-reported hand hygiene compliance increased during the SARS outbreak and remained high nearly 2 years later (Fung & Cairncross, 2007). Higher self-reported compliance in regions of Korea where MERS occurred in 2015 might be interpreted as similar to that during the 2002 SARS outbreak in Hong Kong. To avoid contracting MERS, individuals who lived in MERS outbreak regions were more aware of using soap or hand sanitizer and frequent hand washing than were those who lived outside MERS areas.

The subgroup analyses of hand-washing education revealed that age, sex, income level, and MERS outbreak regions were significantly associated with self-reported hand-washing methods and frequency, although the modifying effects of sex and MERS outbreak regions were not significant. Regarding age, the effect of hand-washing education or promotion on self-reported hand-washing methods and frequency became stronger with increasing age. Generally, older adults have less opportunity to receive education on hand hygiene than do younger generations. Thus, once older adults receive education related to hand hygiene or are exposed to hand hygiene campaigns, their awareness of the importance of hand hygiene may increase. The provision of hand hygiene education or promotion in senior centers might be an effective way to raise older adults’ hand hygiene awareness.

Regarding gender, women who received education about correct hand washing or saw promotional materials related to hand hygiene obtained higher scores for both self-reported use of soap or sanitizer and self-reported hand-washing frequency than did men. Generally, women are more compliant than are men (Lindahl & Heimann, 1997), and they may follow newly learned guidance received through hand hygiene education or promotional materials more easily. Thus, the effect on women of hand-washing education or promotion could be stronger than that on men.

Regarding income level, the effect of hand-washing education or promotion increased as income level decreased. Individuals with relatively lower income have less opportunity to receive education on hand hygiene or be exposed to hand hygiene promotions than do those with higher income. Greater opportunity to receive education on correct hand washing would likely increase their hand hygiene awareness.

Regarding MERS outbreak regions, individuals who lived in MERS outbreak regions and experienced hand-washing education or promotion showed slightly higher scores for self-reported hand-washing frequency than those who did not live in these regions. Additionally, the effect of hand hygiene education or promotion on self-reported use of soap or hand sanitizer when washing hands was similar in individuals who lived in MERS outbreak regions and those who did not. Because the MERS outbreak was a seriously disturbing event, individuals who lived in MERS regions might already have been aware of the importance of washing with soap or hand sanitizer and might have been more likely to do so to avoid getting the disease. Thus, their self-reported behaviors might not have been greatly influenced by hand hygiene education or promotional materials.

The present study has several strengths. First, we used CHS data gathered by a national institution; these data are more statistically reliable than are those from surveys conducted by private survey institutions. Second, our study revealed how specific demographic and socioeconomic factors affected individuals’ hand hygiene behaviors. The present study also has several limitations. First, the CHS is a cross-sectional survey, and we therefore could not establish a causal relationship between hand-washing education and self-reported hand-washing methods and frequency. Second, we used self-reported data to identify hand-washing behaviors, which might have resulted in recall bias. Third, self-reported data of socially desirable behavior such as correct hand washing might have resulted in social desirability bias (Biran et al., 2008; Manun’Ebo et al., 1997). Fourth, careful interpretation of statistical significance is needed due to the very large sample size of this study. Fifth, we could not distinguish between individuals who received hand hygiene education and those who saw promotional materials related to hand washing because the CHS questionnaire asked about both items in a single question. If a later version of the questionnaire could distinguish between hand-washing education and promotion, it might improve our findings regarding the association between hand-washing education and self-reported hand-washing behaviors among Korean adults.

The present study has several implications. Our study indicated that hand-washing education or promotion is important to strengthen individuals’ awareness of correct hand-washing behaviors. Moreover, targeted hand-washing education or promotion could increase individuals’ awareness of hand-washing compliance effectively. Thus, well-organized hand hygiene educational programs or promotional campaigns that address associated demographic and socioeconomic factors we have investigated are needed to prevent infectious diseases. In addition, serious 2015 MERS outbreak in Korea affected individuals’ awareness of proper hand-washing behaviors. Regarding these results, further studies on how long the 2015 MERS effects on hand washing would be sustained and how to keep these positive effects longer are needed to prevent infectious diseases.
